# Medical Thermometry and Human Temperature

**Published:** 1872-03

**Authors:** 


*Medical Thermometer and Human Temperature.* By C. A. WUNDERLICH, M. D., abridged by Edward Seguin, M. D. New York, Wm. Wood, & Co.,

Dr. Seguin in his translation of Prof. Wunderlich's book, has presented the work to us in an abridged form. This abridgement seems to us to contain in a small compass the main points on Medical Thermometry. The need of a work of this character, has long been felt on this side of the Atlantic, and this work will no doubt be received with pleasure.

The translation before us is open to criticism, on account of the foreign idioms used in some instances, and also on account of some portions being included in the abridgement, which might be better supplied from matter left out. Nevertheless it will be gladly received by the American Profession, and will help to a considerable extent to fill up the void in the literarure of this department of medicine. We recommend it to our readers as a valuable addition to their libraries.

